# Etherization Applied to Midwifery

**Published:** 1847-10

**Authors:** J. E. McGirr

**Affiliations:** Chicago


### ARTICLE II.

*Etherization applied to Midwifery.* By J. E. McGirr, A. M., M. D., Chicago.

The application of ether for the purpose of obviating the throes of Parturition, has furnished to eminent European accoucheurs, a theme for discussion, speculation, doubts, and hopes. The celebrated Paul Dubois and Prof. Simpson, deemed it not beneath the dignity of their positions to institute a series of experiments, in order to determine whether the application could be successfully made. These distinguished professors differ in their expectations of the benefits to be derived from it, and, while all are yet in doubt as to its ultimate applicability, a few observations upon the process and Reflex Physiology of Parturition, may afford some ground for basing an opinion. When I say Reflex Physiology of Parturition, I am aware that I am upon debateable ground, such a term not being used in the approved treatises on Obstetrics. But that parturition is clearly a reflex act, the discoveries of Dr. Marshall Hall clearly demonstrate. This subject I intend to consider at some other time. It is by the effects which ether produces upon parturition with reference to this physiological distinction, that the claims of the remedy will be in a great measure decided.

We should carefully examine whether there are any changes, any additions to, or subtractions from the ordinary process of parturition effected by etherization, and then the question will arise, whether such changes, if any, are for good or evil. Etherization is a "new condition of the nervous system," and we should, therefore, note the manner in which the different divisions of that system are affected by the process. These divisions are the cerebral, spinal and ganglionic.

First as to the cerebral, on which the ether soonest and most easily manifests its influence. *Sensation* is temporarily impaired, or abolished. *Volition* is suspended; and though violent and spasmodic actions of the voluntary muscles may take place, they are irregular. *Emotion* also is letheonized.

We have here, then, sensation, volition and emotion suspended, and it becomes therefore necessary, in limine, to consider what are the uses of these acts in the important process of parturition, with which they are so closely allied!

In the last stage of labor, when the motor power is very great and laceration is imminent, sensation and emotion are the safeguards. In this stage, all the actions being reflex, their violence cannot be controlled by the will; and when the pain is almost intolerable, the patient under the influence of emotion, cries out, by which act she opens the glottis, thus taking away all expiratory pressure and leaving the uterus free to act alone. In the last agonies, when the laceration of the perinæum would seem unavoidable, the same cause opens the Sphincter Vesicæ, thereby obviating the danger. Thus much for sensation and emotion, while volition, in the expulsory stage, after the os uteri has been fully dilated, increases the effects of expiration and the power of the abdominal muscles, fixing the thorax and pelvis. It is, therefore, evident that sensation, emotion and volition, are important functions in the process of parturition.

Parturition produces, like a surgical operation, a shock upon the system, and many cases are recorded where death has been evidently the result of this shock. This may occur in any case, even when there is reason to expect a very different result. We have no possible way of avoiding it. And shock is not confined to the brain alone, it is composed of several elements; "those which are really referable to the cerebrum and to the medulla oblongata, which partakes of the nature of brain and spinal marrow—for here the cerebral and spinal systems seem fused—are pain and emotion, and the effects of these depend on the perfect presence of consciousness, and the perception of physical suffering."

Physical pain then is an element of shock in parturition as well as in surgical operations. The severity of the shock is proportioned to the depressing effects of the pain endured.

Emotion constitutes, as observed already, an element of shock. Many persons die from the effects of emotion alone. These two elements of shock, viz: pain and emotion, disappear upon etherization, and it remains to be seen, therefore, whether this partial deliverance is not outweighed by countervailing evils.

The influence of shock upon the spinal marrow, the seat of reflex function and of the ganglionic system, is still greater. An experiment will illustrate this: Remove the contents of the cranium of a frog, leaving the circulation sound; then crush a leg of the animal with the stroke of a hammer, and at once the heart's action ceases, and no irritation can produce reflex action. There being here neither brain nor sensation, the effects of shock upon the medulla spinalis are evident. Again, men have been known to fall suddenly upon the field of battle, desperately wounded, while they were unconscious that there was anything wrong with them. They fell not from pain, nor loss of blood, but from the effects of shock upon the spinal marrow and ganglionic system. Thus their shock affects the spinal marrow independent of sensation or pain, and those that have lauded the use of ether as a means of obviating the shock in operations, have certainly paid no attention to the fact that shock may be produced even in a state of insensibility.

In treating upon the applicability of ether to midwifery, we must notice the therapeutical effects of the remedy upon the brain and spinal marrow. Dr. Ranking found that it increased the spasms of Tetanus. It has been found injurious in hysterical and epileptic seizures, and cases are recorded, in which convulsions resulted from its use. Baron Dubois describes one of the subjects of his experiments, as affected with the most intense premonitory signs of convulsions: "So great," says he, "was the congestion, that I expected her eyes to syringe forth blood." Now, there is no state of the system in which there exists so great a tendency to cerebral congestion, as in that of pregnancy, and weighty indeed should be the reason that could induce any practitioner to add so powerful a stimulant to the utero-spinal excitements of labor.

What benefits then has parturition to expect from etherization, and what dangers has it to fear? Pain and emotion, we have seen, are destroyed; most of the shock that depends upon pain and emotion is no longer felt; but volition, and the salutary influences exerted by pain and emotion upon the motor actions of labor, perish likewise;—

while the whole of those elements of shock that depend upon the spinal marrow and the ganglionic system, are still present in all their intensity. This danger of the physical shock of parturition would thus be added to the shock or collapse occasioned by the ether itself, while the likelihood of convulsions would be much increased. That collapse follows the use of ether has been established by recorded cases, and it is now a settled point that this danger is to be feared even in surgical operations. Dr. W. T. Smith, in his excellent lecture on the inhalation of ether, has noticed several cases. The bare possibility then of producing symptoms, such as stentorous breathing, and pulmonary and cerebral congestion, convulsions, and death by the use of remedial agents, would be enough to make the most daring pause, and consider well what advantages could compensate for so much risk. Strychnia, produces convulsions; opium, cerebral congestion, digitales, retardation of the heart's action—and how carefully does the teacher of therapeutics lay down rules and cautions for our guide in administering these, and yet ether produces the effects of the three; still it is indiscriminately prescribed and lauded, for its safe and certain effects, by those who should know better. It has been argued, that when fatal results have followed, the use of this remedy has been continued too long; but it does not appear from any of the recorded fatal cases, that it was inhaled longer than was necessary to produce insensibility, and in some cases death occurred when there was not entire insensibility during any part of the process.

The important question then, when can ether be safely administered, has yet to be decided.

Baron Dubois communicated, to the Academy of Medicine in Paris, the following results of his experiments on the application of this remedy to parturition. He says:

1st. That the inhalation of ether has the power of preventing pain during obstetric operations.

2d. That it may also momentarily suspend the natural pains of labor.

3d. That the state of ebriety induced by the inhalation of ether does not suspend uterine contraction, when the latter has decidedly set in and takes place at short intervals; and that it does not impede the synergetic action of the abdominal muscles.

4th. The state of ebriety appears to lessen the natural resistance which the perinaeal muscles oppose to the expulsion of the head.

5th. That the inhalation of ether has not appeared to exert any bad influence over the life or health of the child.

Notwithstanding the Baron came to these conclusions, he, after noticing the fatal result of three of his cases, concludes his memoir thus emphatically: "My profound feeling on the subject is, that inhalation of ether in midwifery should be restrained to a very limited number of cases, the nature of which ulterior experience will better allow us to examine."

As to the first conclusion; the application of the forceps, is adduced as one of those operations in which this process is so important. But how much mischief might not an unskilful operator inflict upon a patient whose sensibility was destroyed. The measure of her pain, in such hands, would be the measure of her safety; for if she were not sensible to pain, the os uteri might, just as likely as not, be embraced with the presenting part. While such danger is not imaginary in unskilful hands, the skilful operator needs no such auxiliary to render his success certain and almost painless. So of the other operations. M. Stoltz, of Strasburg, says: "that etherization does not lessen the resistance of the uteras to the introduction of the hand within the cavity, nor does it facilitate the version or expulsion of the child."

As to the third observation: if the uterine contractions continue in all their natural violence, we have already seen the dangers that the suspension of those important auxiliaries to parturient action, viz: pain, emotion and volition, would render almost certain. Such being the facts with respect to two of his conclusions, how comparatively unimportant are the rest!

Who, then, will point out to us the cases, when this important remedy can be safely used to mitigate the sufferings of the parturient! Certainly, no greater boon could be conferred upon mankind. It is no pleasent task to be obliged to treat thus despondingly of a remedy which promised so much that was good; but those who woo science must take her as she presents herself, not as they would have her appear, and truth must be written even against inclination.

It is humiliating to American medical talent, that while the dicovery of the long sought *Letheon* is our own, we must look to Europe, king-ridden Europe, where body and mind are in bondage, for the experiments and physiological facts, that teach how and when safely to apply our own discoveries. This should not be; and I trust the time is not distant when a different organization of the profession will guarantee other results, when European philosophers will look to America as the land of the empire of mind as well as of physical power. Animus homini quic-quid sibi imperat, obtinet.

